# Hormonal Function of Undescended Testes Before Orchidopexy in Prepubertal Boys

**DOI:** 10.3390/jcm14010073

**Published:** 2024-12-27

**Authors:** Renata Walczak-Jędrzejowska, Jerzy Niedzielski, Jolanta Slowikowska-Hilczer, Maciej Nowak, Katarzyna Marchlewska

**Affiliations:** 1 Department of Andrology and Reproductive Endocrinology, Medical University of Lodz, 90-419 Lodz, Poland; jolanta.slowikowska-hilczer@umed.lodz.pl (J.S.-H.); katarzyna.marchlewska@umed.lodz.pl (K.M.); 2 Department of Pediatric Surgery and Urology, University Pediatric Centre, Central University Hospital, Medical University of Lodz, 90-419 Lodz, Poland; jerzy.niedzielski@umed.lodz.pl (J.N.); maciej-roman.nowak@umed.lodz.pl (M.N.)

**Keywords:** undescended testis, intraabdominal testis, testicular volume, testicular atrophy index, testicular growth potential, reproductive hormones

## Abstract

**Background/Objectives:** The hormonal aspect of undescended testes (UDTs) in prepubertal boys, i.e., after mini-puberty, is poorly understood. The aim of this study was to evaluate the hormonal function of UDTs before orchidopexy in prepubertal boys and its correlation with testicular parameters. **Methods:** The study included 90 prepubertal boys (0.9–8.8 years) with UDTs and 57 age-matched boys with testes in the scrotum (control). The testicular volume (TV), testicular atrophy index (TAI), and testicular growth potential (TGP) of the UDTs were assessed before orchidopexy and 18–24 months after. The analysis included FSH, LH, T, DHT, E2, Inh B, INSL3, and AMH levels. **Results**: The UDT group demonstrated a significantly higher FSH level and lower Inh B/FSH ratio than controls. Boys with UDTs aged under six years exhibited significantly higher FSH and LH levels and lower Inh B/FSH and T/LH ratios. The TV level of descended and undescended testes, both before and after surgery, was significantly positively related to T and DHT levels, but negatively with Inh B, INSL3 levels, Inh B/AMH, and Inh B/FSH. **Conclusions:** Hormonal evaluation of the hypothalamus–pituitary–testicular axis and Sertoli and Leydig cell function in prepubertal boys (after mini-puberty) may predict the further development and function of UDTs and may serve as a diagnostic tool in testicular descent disorder.

## 1. Introduction

Undescended testis (UDT) is a condition where a male gonad is located outside the scrotum. It is typically palpable in the inguinal canal, but in approximately 6% of cases, it is not [1,2], in which case, it may be either intra-abdominal (IAT) or absent (agenesis or vanishing testis) [1,3].

UDT can be evaluated, and future testis function predicted, based on testicular volume (TV), testicular atrophy index (TAI), and testicular growth potential (TGP) [4,5]. A significant decrease in TV and an increase in TAI are observed in patients with a high located UDT and treated later during childhood [1,2,6,7,8,9].

UDT may occur as a consequence of primary testicular dysfunction (gonadal dysgenesis), a central disorder of the gonadotropin-releasing hormone (GnRH)–gonadotroph axis, or an anatomic malformation independent of any endocrine disorder [10,11]. Hormones such as anti-Müllerian hormone (AMH) and inhibin B (Inh B), as well as follicle-stimulating hormone (FSH) (indirectly), serve as markers of Sertoli cell function. Sertoli cells act as “nurse cells” for germ cells in the testes, playing a crucial role in spermatogenesis within the seminiferous tubules. Similarly, insulin-like peptide 3 (INSL3), testosterone (T), and luteinizing hormone (LH) (indirectly) are markers of Leydig cell function, which are located in the testicular interstitium. Understanding testicular hormonal function at the time of UDT diagnosis provides insight into the pathophysiology of the condition. Additionally, it may be useful in evaluating treatment outcomes and predicting hormonal secretion during puberty and adulthood, as well as fertility potential. It has been suggested that routine endocrine and genetic assessments could become an effective component of clinical management for boys with UDTs [12,13]. However, the current algorithm for the urological management of UDT does not incorporate hormonal diagnostics.

This study aims to evaluate the serum concentrations of testicular and pituitary hormones shortly before orchidopexy in prepubertal boys and to correlate these findings with TV, TAI, and TGP, thereby predicting the future development of UDT.

## 2. Materials and Methods

The study was conducted in accordance with the Declaration of Helsinki and approved by the Bioethics Committee of the Medical University of Lodz (No RNN/43/15/KE of 17 March 2015).

Informed consent to conduct clinical evaluation and blood collection for hormonal measurements was obtained from the parents or guardians of all participants.

### 2.1. Patients

Included in the study were 90 prepubertal boys (aged 0.9–8.8 years) with congenital UDT visiting the Surgical Clinic of the Ambulatory Care Unit of the University Pediatric Centre, Central University Hospital of Medical University in Lodz, Poland, between May 2020 and April 2023. The reference group (control group) consisted of 57 prepubertal age-matched boys (1.0–9.9 years) with testes in the scrotum who underwent surgery due to inguinal hernia.

Surgery was performed at 1.0–8.8 years of age in boys with unilateral canalicular UDT (UCT; N = 65), at 1.2–8.7 years in those with bilateral canalicular UDT (BCT; N = 13), at 0.9–2.0 years in those with intra-abdominal testes (IAT, N = 12), and at 1.0–9.9 years in those with inguinal hernia (N= 57). None of the boys received hormonal therapy.

Each patient underwent a physical examination according to the Tanner’s scale; the size of each testis was measured with a Prader’s orchidometer. In each UDT patient, the position and size of the gonad were also assessed by ultrasonography before and 18–24 months after the surgical treatment. All the patients were otherwise healthy.

Samples for hormonal determinations were drawn from all patients one day before the surgical treatment.

The boys were considered to be prepubertal when they met criteria of stage 1 of the Tanner’s scale (TV < 4.0 cm^3^). The patients were also divided into age subgroups based on the developmental changes in their testes, as follows: 0.9–5.9 (<6th year) and 6.0–9.9 (≥6th year) of age [14]. The UDT patients were further subdivided into UCT, BCT, or IAT groups

### 2.2. Scrotal Ultrasonography

In patients with UDT, the position and three dimensions of both testes were recorded by the means of ultrasonography and used to calculate the testis volume, TV, as follows: TV (cm^3^) = 0.72 × width (cm) × length (cm) × height (cm). The result was used to calculate the TAI of the affected testis, as follows: TAI (%) = [(contralateral TV − affected TV)/contralateral TV] × 100% [4]. The TAI of BCTs was calculated assuming the median TV of healthy gonads of the UCTs of the appropriate age group as a reference. TAI-1 refers to the undescended testes in the UCT and IAT groups and larger testes in BCT group compared to the mean TV of the healthy testes in the UCT group. TAI-2 refers to the smaller testes in the BCT group compared to the mean TV of the healthy testes in the UCT group.

TGP was calculated as the percentage difference in TV measured before (B) and after (A) surgery, as follows: TGP (%) = [(TVA − TVB)/TVB] × 100%. TGP-1 means descended testes in the UCT and IAT groups and bigger testes in the BCT group, and TGP-2—undescended testes in the UCT and IAT groups and smaller testes in the BCT group.

### 2.3. Blood Samples and Hormone Measurements

In each patient, a fasting blood sample was drawn from the cubital vein in the morning hours (between 7:00 and 10:00 am) and centrifuged. Serum was then separated and kept frozen at −80 °C until hormone analysis was performed.

All hormone levels were measured using commercially available kits according to the manufacturer’s protocol. The serum levels of FSH, LH, T, and estradiol (E2) were determined using the enhanced chemiluminescence method on a VITROS ECi Immunodiagnostic System with MicroWell technology (Ortho-Clinical Diagnostics, Addlestone, UK). The respective intra- and inter-assay coefficients of variations (CV) were <2.8% and <10.6% for measurements of FSH, <8.8% and <12.8% for LH, <3.8% and <7.4% for T, and <6.6% and <8.8% for E2. The limit of detection was 0.66 IU/L for FSH, 0.21 IU/L for LH, 0.17 nmol/L for T, and 23 pmol/L for E2. Inh B and AMH serum concentrations were determined using enzyme-linked immunoabsorbent assay (AnshLabs, Webster, TX, USA). The respective intra- and inter-assay CV values were <4.9% and <6.3% for Inh B and <4.0% and <4.8% for AMH. The limit of detection was 1.6 pg/mL for Inh B and 0.06 ng/mL for AMH. Dihydrotestosterone (DHT) levels were determined by the competitive enzyme immunoassay (Labor Diagnostika Nord GmbH & Co. KG (LDN), Nordhorn, Germany). Intra- and inter-assay CV were <11.4% and <12.1% for DHT, respectively, and the limit of detection was 6 pg/mL. INSL3 levels were measured using the sandwich enzyme-linked immunoassay (Cloud-Clone Corp., Katy, TX, USA). The intra- and inter-assay CV for INSL3 were <10% and <12%, respectively, and the limit of detection was 5.0 pg/mL.

For the undetectable results, the value corresponding to the limit of detection for a specific hormone was used in the statistical analysis. The following hormone reference values were adopted for prepubertal boys: T—<0.5 nmol/L, DHT—<30 pg/mL, E2—<23–34 pmol/L, Inh B—35–182 pg/mL, AMH—55–187 ng/mL, INSL3—30.0–70.0 pg/mL, FSH—<0.66–1.6 U/L, and LH—<0.5 U/L [15,16,17,18].

The ratios of Inh B/FSH, AMH/FSH, and INSL3/LH were calculated.

### 2.4. Statistics

All statistical analyses were performed using Statistica 13.1 for Windows (StatSoft Inc., Tulsa, OK, USA). The distribution of the data was determined using the Shapiro–Wilk test. As the data demonstrated a non-normal distribution, they are presented as mean ± SD, median, and interquartile range and the groups were compared using the ANOVA, Kruskall–Wallis, or Mann–Whitney tests as appropriate. Spearman rank correlation coefficient (rs) was applied to examine the relationships between the analyzed parameters. Differences were considered significant at *p <* 0.05.

## 3. Results

Among the whole group of prepubertal boys, the T concentration was below the limit of detection in 73%, FSH in 35%, and LH in 59%. The control and study groups had similar percentages of undetectable results for each hormone (FSH—40% vs. 31%, LH 63% vs. 56%, and T 73% vs. 74%).

Intergroup comparisons with regard to age and hormone level are shown in Table 1. The control group had a higher mean age than the UDT groups. The mean age of boys with UCT and IAT was significantly lower than that of the boys in the control group. The entire group of UDT boys demonstrated a significantly higher FSH serum level and lower Inh B/FSH ratio compared to controls. No significant differences in hormone levels were found between the UDT groups.

Regarding the age subgroups, lower LH and Inh B levels and a lower Inh B/AMH ratio was observed in older UDT boys compared to age-matched controls, as well as a higher T/LH ratio (Table S1a, Supplementary Materials). In contrast, younger UDT boys exhibited significantly higher FSH and LH levels, along with lower Inh B/FSH and T/LH ratios compared to controls.

In the control group, the older boys exhibited an increase in FSH, LH, T, and DHT levels, along with a decrease in the INSL3/LH ratio compared to younger boys. In the UDT group, older boys showed higher T and DHT levels and a lower T/LH ratio compared to younger boys, while Inh B, INSL3, and, unexpectedly, LH levels were higher.

In the control group, age exhibited significant positive correlations with FSH, LH, T, and E2 and negative correlations with AMH (Table S1b, Supplementary Materials). Conversely, in UDT boys, age demonstrated significant positive correlations with T and DHT and negative correlations with Inh B and INSL3.

The testicular parameters of the UDT subgroups are given in Table 2. As expected, in prepubertal boys, the youngest IAT group had a significantly lower TV of descended and undescended testes before surgery in comparison to the UCT and BCT groups; however, after surgery, significant differences were only observed between the IAT and UCT groups. In the BCT group, TAI-1 (larger testis) was the lowest only before surgery. Significantly higher TGPs were found for both undescended and descended testes in the IAT group.

The testicular parameters of boys with UDT below six years and those above are compared in supplementary Table S2a (Supplementary Materials). The older group demonstrated higher TV-1, TV-2, and, thus, mean TV before and after surgery; they also exhibited significantly lower TGP-1, TGP-2, and mean TGP. Only TAI-1 was significantly lower at the beginning of the observation period.

As expected, when analyzing the associations between age and testicular parameters in the entire UDT group, a significant positive correlation was found between age and TV for both descended and undescended testes, both before and after surgery. Additionally, age was negatively correlated with TGP and with TAI-1, but only before surgery. Similar correlations were noted in the UCT group (except for TAI-1 before surgery) and the BCT group (except for TGP and TAI-1 before surgery). In the IAT group, the only correlations observed were between age and mean TV before and after surgery, as well as TV-1 after surgery (Table S2b, Supplementary Materials).

Among the prepubertal boys with UDT, the TV of descended and undescended testes both before and after surgery was significantly positively related to T and DHT, but negatively related to Inh B and INSL3 levels, as well as the Inh B/AMH and Inh B/FSH ratios (Table 3). TAI was not significantly related to any hormone before and after surgery. TGP was correlated negatively with DHT and positively with Inh B. Additionally, only TGP-2 was related positively to the Inh B/AMH ratio.

Generally speaking, the majority of correlations between hormone levels and the testicular parameters observed in the whole UDT group were also found in the UCT group, but not in the BCT or IAT groups. In addition, in the UCT group, a positive correlation was noted between the TV of descended and undescended testes, before or after surgery, and the T/LH ratio (Table S3a, Supplementary Materials).

In the boys under six years of age, only TV-1 correlated negatively with Inh B and Inh B/FSH before and after surgery; no such correlation was visible in older boys (Table S3b, Supplementary Materials). In boys aged six years or above, TV-1 was correlated positively with T and TV-2 was correlated positively with AMH before and after surgery; however, TV-1 was correlated with the T/LH ratio only after surgery. TAI was not correlated with any hormone at any time of the observation. Only TGP-2 was correlated significantly with the Inh B/AMH ratio in younger boys.

TV-1 and TV-2 were negatively correlated with INSL3 levels before and after surgery, in the whole UDT group. It is worth noting that, this negative correlation, although non-significant, was observed only in the group of younger boys; a positive correlation was noted among the older boys. More specifically, in the younger UDT boys, the following correlations were identified: between TV-1 (B) and INSL3 levels, rs = −0.21, *p* = 0.09; between TV-2 (B) and INSL3 levels, rs = −0.06, *p* = 0.65; between TV-1 (A) and INSL3 levels, rs = −0.18, *p* = 0.16; and between TV-2 (A) and INSL3 levels, rs = −0.07, *p* = 0.59. In older boys, the following correlations showed a positive trend: between TV-1 (B) and INSL3 levels, rs = 0.365, *p* = 0.21; between TV-2 (B) and INSL3 levels, rs = 0.06, *p* = 0.65; between TV-1 (A) and INSL3 levels, rs = 0.31, *p* = 0.29; and between TV-2 (A) and INSL3 levels, rs = 0.04, *p* = 0.89.

Table 4 presents the correlations between the studied hormones in the control and UDT groups. In the control group, FSH showed a negative correlation with AMH, while LH exhibited positive correlations with all steroid hormones (T, DHT, and E2). Moreover, T significantly correlated with both of its metabolites. Additionally, E2 showed a positive correlation with AMH. In contrast, in the UDT group, positive correlations were observed between LH and FSH, as well as between LH and Inh B. Furthermore, Inh B was positively correlated with INSL3 and T was correlated positively with only one of its metabolites, DHT.

Supplementary Table S4a,b present the correlations between hormones in the control and UDT age subgroups. In the control group, a negative correlation was observed between FSH and AMH in younger boys (under six years), but not in older ones. Conversely, correlations between LH and steroid hormones, as well as between the steroid hormones themselves, were found exclusively in older boys. In younger boys, LH was correlated only with T and E2, and no correlations were observed among the steroid hormones. Additionally, a positive correlation between Inh B and INSL3 was observed only in younger boys. In the control group, E2 consistently showed a positive correlation with AMH in both age subgroups.

In the UDT group, LH correlated positively with FSH and INSL3, but these correlations were limited to the younger group. In contrast, LH correlated with Inh B and AMH in the older boys. Among steroid hormones, the only correlation consistently observed across both UDT age subgroups was between T and DHT. Similarly to the control group, E2 showed a positive correlation with AMH, but only in younger boys. Similarly to the control group, a positive correlation between Inh B and INSL3 was also observed in younger UDT boys. Additionally, a correlation between Inh B and AMH was observed exclusively in older UDT boys.

In the prepubertal boys with UDT, compared to reference values, 16% had elevated FSH (UCT—15%, BCT—15%, and IAT—17%), 13% had elevated LH (UCT—17%, BCT—8%, and IAT—1%), and 11% had elevated FSH or LH (UCT—12%, BCT—15%, and IAT—0%). Furthermore, the AMH level was elevated above the reference values in 5% of UDT boys (UCT—4.6%, BCT—15%, and IAT—0%) and the Inh B level in 15.5% of them (UCT—18.5%, BCT—0%, and IAT—17%). In turn, the AMH levels were lower than the reference values in 25.5% of the boys with UDT (UCT—23%, BCT—38.5%, and IAT—25%) and the Inh B levels in 12% of them (UCT—8%, BCT—8%, and IAT—8%). In addition, 4% of UDT boys demonstrated lower levels of both AMH and Inh B (UCT—5%, BCT—8%, and IAT—0%).

## 4. Discussion

The testis may fail to descend due to various adverse factors. Among others, disturbed testicular development and, for this reason, poor function, is worth diagnosing in childhood in order to predict its hormonal and seminiferous activities during puberty and in adulthood. The best period for performing tests aimed at determining the function of undescended testes is during mini-puberty, i.e., two to three months after birth, when the hypothalamus–pituitary–gonadal (HPG) axis is active and it is possible to detect some testicular disorders. However, the hormonal evaluation of boys with UDT is usually not performed or performed much later, when the boy is submitted to testicular surgery. The present study examines whether it is possible to identify any disorders of testicular function in prepubertal boys with UDT older than six months, i.e., after mini-puberty.

### 4.1. Hypothalamus–Pituitary–Gonadal Axis Function

The period from mini-puberty to puberty is considered to be “silent”, i.e., one when the HPG axis is not active [19]. However, it has been noted that, even in this period of life, an elevation of gonadotropin levels associated with primary gonadal failure is first manifested by an increased FSH serum concentration, which is not clearly regulated by gonadal steroids [20,21,22,23,24]. Our present findings indicate a significantly higher mean serum level of FSH in boys with UDT in comparison to controls, i.e., boys with descended testes. It was particularly visible in boys below six years of age. Their LH level was also increased, but not significantly, probably because the levels were not detectable in almost 60% of cases. In the UDT group, 11% had increased FSH and LH, indicating possible damage to the testes. Such a situation could be connected with a lower inhibin B level, as noted by other authors [21,23].

### 4.2. Sertoli Cell Function

In the present study, the Inh B level was decreased, but not significantly, in the whole UDT group, and significantly so in boys six years of age or older. However, the Inh B/FSH ratio was significantly lower in the UDT group than in controls. Inhibin is produced exclusively by the Sertoli cells of the testes, and its serum level varies in response to FSH secretion. Any disorder in Sertoli cell function can lower the Inh B level. Sertoli cells are extremely important for the survival and proper development of spermatogenesis, thus, the serum values of Inh B reflect the state of the seminiferous epithelium in cryptorchid testes.

Another hormone secreted by the Sertoli cells in childhood is AMH [17,25]. After birth, AMH increases significantly during the infantile period of mini-puberty; later, at the end of the first year, it declines and remains at a relatively stable level until the time of puberty. At the onset of puberty, its secretion declines in parallel with Sertoli cell maturation and the increasing secretion of T from the Leydig cells. AMH is a marker of the normal development of testes in newborn infants with ambiguous genitalia or nonpalpable gonads [26]. Rey et al. [14] showed that AMH was low in children with disorders/differences in sex development (DSD) caused by abnormal testicular determination (pure or partial gonadal dysgenesis), but was normal or elevated in patients with impaired T secretion; in addition, both groups demonstrated low serum T. In the present study, 25.5% of boys from the UDT group exhibited AMH levels below the lower reference limit; however, only 0.04% were found to have low AMH and low Inh B levels, suggesting Sertoli cell dysfunction. None of the patients presented undetectable AMH, which is highly suggestive of anorchia. A previous study by Szarras-Czapnik et al. [27] found the AMH levels to be normal in 50% of prepubertal boys with UDTs. A similar prevalence of normal AMH levels was noted in our study (50%), and none of the AMH levels in any of the various UDT groups differed significantly from those of the control group. However, the remaining 50% of boys had higher or lower levels than the reference values, indicating the heterogeneity of UDT function.

Furthermore, higher levels of AMH were noted in the IAT group, but these differences were not significant. This may suggest that the etiology of some UDTs in this group may be associated with a lack of T action. It is known that the lack of androgen receptor expression in Sertoli cells could account for a physiological androgen insensitivity during the early prepubertal period of life; this, in turn, may serve to protect Sertoli cells from precocious maturation, resulting in proliferation arrest and spermatogenic development [28]. The number of androgen receptors increases with the advancing age until puberty. Rey et al. [13,14] reported elevated AMH levels and low serum T levels in children with ambiguous genitalia and androgen synthesis defects or androgen insensitivity syndrome. Higher levels of AMH were detected in 5% of UDT boys, suggesting that the reason that the testes did not descend in these boys may have been the disturbed androgen action.

A significant positive correlation was observed between the levels of E2, the metabolite of T, and the AMH produced by immature Sertoli cells. Studies of fetal and immature animals indicate that Sertoli cells are the main source of estrogens, and that the expression of aromatase in Leydig cells is very low at this time [29,30]. Our present data indicated higher E2 levels in UDT boys, similarly to the AMH levels, but not significantly. This may be the result of stronger Sertoli cell stimulation by higher FSH levels in some children.

### 4.3. Leydig Cell Function

INSL3 is produced by testicular Leydig cells. It is involved in the intra-abdominal descent of testes to the scrotum during the prenatal period of life. Disorders in INSL3 function, such as mutations in *INSL3* or the *RXFP2* receptor, may result in cryptorchidism [31]. It was found that INSL3 may regulate male germ cell survival [32]; however, its role in the regulation of testicular function postnatally is unclear. The serum INSL3 concentration is considered as a diagnostic marker for Leydig cell maturity and function in addition to T level [33]. Albrethsen et al. [34] revealed that the INSL3 level increases during puberty from almost undetectable levels in boys aged 5–10 years; these levels rise in parallel with changes in T. In our study, the levels of INSL3 were low in prepubertal boys, and no significant differences were found between the UDT and control groups. However, a positive correlation was found between the INSL3 and LH levels, which was predictive. Other authors [35] suggest that INSL3 secretion may be dependent on the differentiating effect of LH on Leydig cells, but independent of the steroidogenic LH-mediated action. Thus, even though T and INSL3 are both dependent on LH, these two Leydig cell hormones are regulated differently.

INSL3 also demonstrated a significant positive correlation with Inh B. This relationship may be explained by the results of Raivio et al. [22], who observed an increase in the serum Inh B concentration during hCG stimulation in prepubertal boys aged from one to eight years with cryptorchidism. The authors attributed this to the indirect stimulation of Sertoli cells via Leydig cells. A relationship between Sertoli and Leydig cells in prepubertal testis was noticed by Racine et al. [36]. They found that the AMH receptor is expressed in Leydig cells and suggest that AMH may modulate Leydig cell numbers and function by controlling the differentiation of mesenchymal precursors and the expression of steroidogenic enzymes in the immature testis.

The levels of T did not differ between the UDT and control groups. The levels of DHT, the testosterone metabolite, were lower in UDT group, but not significantly, in comparison with the control group. However, a significant positive correlation was noted between T and DHT in prepubertal boys. Taken together, these data indicate a poorer production of T by the UDT group. The DHT level may serve as an indirect marker of T production in the testes of prepubertal boys, as the T level is usually below the reference value.

### 4.4. Parameters of Testicular Growth

Previous research indicates that UDTs undergo continuous growth until puberty, regardless of their position, and that IATs possess a high growth potential, although less than that of UCTs and BCTs [9]. Our present findings confirm these results. The better testicular growth potential of IATs may be explained by the lower T activity compared to other UDT groups, i.e., lower DHT levels and higher AMH levels, which may intensify Sertoli cell proliferation and delay their maturation.

For us, the hormonal indicators of UDT growth observed after surgery were of particular interest. More specifically, positive correlations were noted between TGP and Inh B, as well as DHT. This confirms that better Sertoli and Leydig cell functions predict a better growth of UDTs.

Unexpectedly, a negative correlation was noted between the INSL3 levels and TV of descended, potentially healthy testes, and those of the boys with UDT before surgery. There are currently no such data on this phenomenon in the literature. Indeed, previous studies on healthy boys [34,37,38] demonstrated that the INSL3 levels increased in parallel with TV as age advanced; however, these studies included older subjects than those in the present study, ranging from 9.5 to 17.5 years. In contrast, 66 boys with UDT in our study were under the age of five years. Moreover, in the present study, the negative correlations between hormones and testicular growth parameters persisted only among the younger boys; they shifted to positive in the older boys, although neither correlation was statistically significant. Of course, it is possible that this negative correlation may have occurred by chance, especially given the large inter-individual variation in the INSL3 levels among the subjects. Therefore, further studies are needed to confirm this observation and, if validated, to elucidate the underlying mechanisms and the role of INSL3 in the development of the male gonad.

TAI turned out not to be a good predictor of testicular hormonal function, because it did not correlate with any hormone.

### 4.5. Strengths and Limitations of the Study

A key strength of our study is its hormonal evaluation, which provides an insight into the testicular function in prepubertal boys with UDT; the results may be predictive of testicular development and function in adulthood. Similar studies are scarce in the existing literature. However, it is important to note the relatively low number of patients included in the study, which may limit the statistical analysis.

We used a control group of age-matched boys with inguinal hernia, as this was the only feasible group for justifying repeated blood sampling in young children. However, this control group is not ideal for comparisons with boys with UDTs, as both conditions may be congenital and could share some similarities in etiology [39]. Additionally, evidence suggests that both cryptorchidism and inguinal hernia are associated with an increased risk of testicular germ cell cancer [40,41]. This raises the possibility of a common underlying cause for some cases of UDT and inguinal hernia, potentially involving hormone-regulated pathways. However, further research is needed to clarify this connection.

## 5. Conclusions

Although prepubertal boys may be treated for UDTs, they typically do not undergo hormonal evaluation of the HPG axis or assessments of their Sertoli and Leydig cell function following mini-puberty. Our findings underscore the clinical importance of performing such evaluations for improved management. Elevated serum FSH levels, accompanied by low levels of Inh B and AMH, may signal Sertoli cell dysfunction and disruptions in spermatogenesis, potentially indicating a reduced fertility potential in adulthood. Similarly, increased LH levels, combined with low T and DHT levels, may suggest Leydig cell dysfunction and the potential need for testosterone replacement therapy during puberty.

The measurement of serum DHT concentration may serve as an indirect marker of T production in the testes of prepubertal boys, as T levels are often below the reference range at this age. The absence of hormonal indicators of testicular damage, even in prepubertal boys, is a positive prognostic factor for the growth of UDT and suggests favorable testicular function. Furthermore, comparing the hormonal activity of the testes before and after orchidopexy provides valuable insight, alongside testicular volume growth, into the efficacy of the surgical intervention.

Hormonal evaluations in prepubertal boys may also help to identify the underlying causes of testicular descent disorders, such as gonadal dysgenesis, disorders of androgen synthesis, androgen insensitivity, or disruptions in AMH and INSL3 production or action. Early identification of the cause can guide subsequent medical management and necessitate closer monitoring of these patients, as certain disorders—such as gonadal dysgenesis and androgen insensitivity—are associated with an increased risk of testicular germ cell tumors.

## Figures and Tables

**Table 1:** Comparisons of hormone levels between all boys with undescended testes (UDT) and groups with different clinical types of UDT: unilateral canalicular (UCT), bilateral canalicular (BCT), and intra-abdominal (IAT).

Parameters	Control N = 57	UDT N = 90	UDT Groups		
			UCT N = 65	BCT N = 13	IAT N = 12
	Mean ± SD Median Min–Max	Mean ± SD Median Min–Max	Mean ± SD Median Min–Max	Mean ± SD Median Min–Max	Mean ± SD Median Min–Max
Age (years)	5.3 ± 2.5 5.0 1.0–9.9	**3.8 ± 2.5 ^a^** **3.1** **0.9–8.8**	**4.0 ± 2.3 ^b^** **3.6** **1.0–8.8**	4.7 ± 2.4 3.9 1.2–8.7	**1.4 ± 0.4 ^c,d,e^** **1.3** **0.9–2.0**
FSH (U/L)	1.0 ± 0.4 0.8 0.7–2.7	**1.3 ± 0.7 ^a^** **1.0** **0.7–4.1**	1.3 ± 0.8 1.0 0.7–4.1	1.2 ± 0.7 1.0 0.7–2.7	1.0 ± 0.5 0.6 0.7–2.2
LH (U/L)	0.3 ± 0.3 0.2 0.2–1.7	0.4 ± 0.4 0.2 0.2–2.1	0.4 ± 0.3 0.3 0.3–2.0	0.5 ± 0.5 0.2 1.2–8.7	0.2 ± 0.0 0.2 0.2–2.0
T (nmol/L)	0.2 ± 0.1 0.2 0.2–0.8	0.2 ± 0.1 0.2 0.2–0.7	0.2 ± 0.2 0.2 0.2–0.7	0.2 ± 0.1 0.2 0.2–0.4	0.2 ± 0.0 0.2 0.2–0.3
E2 (pmol/L)	40.6 ± 15.4 36.9 23.0–99.6	44.6 ± 18.2 41.7 23.0–105.2	43.9 ± 17.6 40.5 23.0–102.8	40.4 ± 18.5 36.5 23.0–80.4	51.9 ± 20.9 48.1 31.6–105.2
DHT (pg/mL)	134.9 ± 134.9 73.0 9.0–500.0	97.8 ± 101.4 61.0 10.0–469.0	102.0. ± 100.0 64 11.0–469.0	109.6 ± 134.8 47.0 11.0–438.0	53.2 ± 49.4 36.0 10.0–174.0
Inh B (pg/mL)	126.1 ± 77.2 126.7 3.1–344.4	110.1 ± 71.1 99.1 6.7–322.9	114.4 ± 75.5 99.7 6.7–322.9	84.2 ± 36.9 93.1 10.2–146.7	119.4 ± 76.3 122.3 10.1–222.1
AMH (ng/mL)	87.5 ± 71.1 64.0 13.0–240.0	94.9 ± 77.4 82.0 12.6–335.0	93.2 ± 77.4 82.0 12.6–335.0	84.9 ± 76.6 71.0 14.9–234.0	121.1 ± 82.3 146.0 15.7–222.0
INSL3 (pg/mL)	16.6 ± 12.6 11.7 5.6–75.0	18.0 ± 7.8 19.4 5.8–32.1	18.2 ± 7.9 19.2 5.8–32.1	16.2 ± 7.6 18.8 5.7–26.3	19.4 ± 8.1 21.0 6.6–29.3
T/LH	0.8 ± 0.2 0.8 0.2–1.7	0.7 ± 0.4 0.8 0.1–3.0	0.7 ± 0.5 0.8 0.1–3.0	0.7 ± 0.5 0.8 0.1–2.0	0.9 ± 0.1 0.8 0.8–1.2
Inh B/FSH	143.9 ± 110.7 113.2 4.7–521.8	**114.8 ± 110.7 ^a^** **80.0** **4.1–594.9**	120.5 ± 120.0 86.8 4.1–594.9	77.7 ± 49.6 62.8 15.5–203.8	130.2 ± 101.2 119.5 15.3–330.5
AMH/FSH	106.0 ± 99.8 90.9 5.2–363.6	92.5 ± 88.2 76.5 6.1–384.4	89.3 ± 85.9 67.9 6.1–384.4	82.4 ± 88.5 78.0 7.0–337.5	127.9 ± 103.8 123.3 17.6–313.7
Inh B/AMH	3.7 ± 4.5 1.4 0.1–18.7	2.9 ± 4.1 1.0 0.1–23.4	3.0 ± 04.4 1.1 0.1–23.4	2.2 ± 2.4 0.9 0.5–6	2.7 ± 3.8 1.0 0.1–10.9
INSL3/LH	63.2 ± 54.9 43.2 4.9–347.2	59.6 ± 36.3 48.6 4.9–148.1	58.1 ± 34.1 46.4 4.9–148.1	48.4 ± 30.7 36.1 11.8–98.2	89.9 ± 37.7 97.2 30.1–135.6

*p* < 0.05, ^a^—control vs. UDT; U Mann–Whitney test; ^b^—control vs. UCT; ^c^—control vs. IAT; ^d^—UCT vs. IAT; ^e^—BCT vs. IAT; ANOVA Kruskal–Wallis test. Statistically significant data are in bold. Abbreviations: AMH—anti-Mȕllerian hormone; DHT—dihydrotestosterone; E2—estradiol; FSH—follicle-stimulating hormone; Inh B—inhibin B; INSL 3—insulin like protein 3; LH—luteinizing hormone; N—number of cases; T—testosterone.

**Table 2:** Comparisons of testicular parameters between groups with different clinical types of undescended testes: unilateral canalicular (UCT), bilateral canalicular (BCT), and intra-abdominal (IAT).

Testicular Parameters	UCT N = 65	BCT N = 13	IAT N = 12
	Mean ± SD Median Min–Max	Mean ± SD Median Min–Max	Mean ± SD Median Min–Max
Unilateral n (%) Bilateral n (%)	65 (100%) -	- 13 (100%)	12 (100%) -
TV-1 (B)	0.9 ± 0.4 0.9. 0.3–2.2	0.8 ± 0.3 0.8 0.3–1.4	**0.4 ± 0.1 ^b,c^** **0.4** **0.3–0.5**
TV-2 (B)	0.7 ± 0.3 0.7 0.2–1.7	0.7 ± 0.3 0.7 0.2–1.3	**0.2 ± 0.1 ^b,c^** **0.2** **0.1–0.4**
Mean TV (B)	0.8 ± 0.4 0.8 0.3–2.0	0.7 ± 0.3 0.7 0.3–1.4	**0.3 ± 0.1 ^b,c^** **0.3** **0.2–0.5**
TAI-1 (B)	23.6 ± 13.6 22.2 0–68.7	**13.6 ± 12.6 ^a,c^** **12.5** **0–48.9**	34.6 ± 25.5 41.7 0–75.0
TAI-2 (B)	-	33.2 ± 21.3 23.8 13.3–74.4	-
TV-1 (A)	1.2 ± 0.5 1.1 0.5–3.5	1.1 ± 0.4 1.0 0.7–2.0	**0.8 ± 0.1 ^b^** **0.8** **0.6–1.0**
TV-2 (A)	1.0 ± 0.4 0.9 0.4–2.9	0.9 ± 0.5 0.7 0.1–2.0	**0.6 ± 0.1 ^b^** **0.6** **0.4–0.8**
Mean TV (A)	1.1 ± 0.5 1.0. 0.5–3.2	1.0 ± 0.4 0.9 0.5–2.0	**0.7 ± 0.1 ^b^** **0.7** **0.5–0.9**
TAI-1 (A)	17.2 ± 12.2 16.7 0–63.2	13.3 ± 10.4 12.1 −5.3–27.5	21.2 ± 13.1 20.0 0–37.5
TAI-2 (A)	-	31.7 ± 30.1 28.3 −0.8–88.3	-
TGP-1	40.0 ± 37.0 25.0. 0.0–200.0	40.9 ± 51.1 22.5 4.4–156.5	**109.7 ± 47.2 ^b,c^** **100.0** **50.0–200.0**
TGP-2	54.7 ± 47.1 42.9 −28.6–200.0	33.6 ± 51.5 29.2 −54.8–170.4	**169.1 ± 64.2 ^b,c^** **156.3** **75.0–300.0**
Mean TGP	45.7 ± 40.2 34.5 −13.3–200.0	39.5 ± 45.1 22.3 −5.6–159.6	**125.9 ± 37.4 ^b,c^** **116.7** **66.7–200.0**

*p* < 0.001, ^a^—UCT vs. BCT, ^b^—UCT vs. IAT, ^c^—BCT vs. IAT; ANOVA Kruskal–Wallis test; N—number of cases. Statistically significant data are in bold. Abbreviations: A—after surgery; B—before surgery; TAI—testicular atrophy index (%); TAI-1—undescended testes in UCT and IAT group, bigger testes in BCT group compared to the healthy testes in UCT group; TAI-2—smaller testes in BCT group compared to the healthy testes in UCT group; TGP—testicular growth percentage (%); TGP-1—descended testes in UCT and IAT group, larger testes in BCT group; TGP-2—undescended testes in UCT and IAT group, smaller testes in BCT group; mean TGP—mean of both testes; TV—testicular volume (cm^3^); TV-1—descended testis in UCT and IAT groups, bigger testis in BCT group; TV-2—undescended testis in UCT and IAT groups, smaller testis in BCT group; mean TV—mean of both testes.

**Table 3:** Spearman’s rank correlations (rs) between testicular parameters and serum hormone levels in boys with UDT.

Parameter	N	TV-1 (B)	TV-2 (B)	Mean TV (B)	TAI-1 (B)	TAI-2 (B)	TV-1 (A)	TV-2 (A)	Mean TV (A)	TAI-1 (A)	TAI-2 (A)	TGP-1	TGP-2	Mean TGP
FSH	90	NS	NS	NS	NS	NS	NS	NS	NS	NS	NS	NS	NS	NS
LH	90	NS	NS	NS	NS	NS	NS	NS	NS	NS	NS	NS	NS	NS
T	90	0.31 **	0.22 *	0.29 **	NS	NS	0.32 **	0.26 *	0.32 **	NS	NS	NS	NS	NS
E2	90	NS	NS	NS	NS	NS	NS	NS	NS	NS	NS	NS	NS	NS
DHT	85	0.34 **	0.34 **	0.35 ***	NS	NS	0.23 *	0.24 *	0.26 *	NS	NS	−0.27 *	−0.22 *	−0.26 *
Inh B	83	−0.39 ***	−0.32 **	−0.38 ***	NS	NS	−0.34 **	−0.23 *	−0.30 **	NS	NS	0.26 *	0.27 *	0.24 *
AMH	83	NS	NS	NS	NS	NS	NS	NS	NS	NS	NS	NS	NS	NS
INSL3	76	−0.34 **	−0.24 *	−0.30 **	NS	NS	−0.30 **	NS	−0.25 *	NS	NS	NS	NS	NS
T/LH	90	NS	NS	NS	NS	NS	0.24 *	NS	0.22 *	NS	NS	NS	NS	NS
Inh B/FSH	83	−0.31 **	−0.29 *	−0.32 *	NS	NS	−0.30 **	−0.28 *	NS	NS	NS	NS	NS	NS
AMH/FSH	83	NS	NS	NS	NS	NS	NS	NS	NS	NS	NS	NS	NS	NS
Inh B/AMH	83	−0.26 *	−0.27 *	−0.28 **	NS	NS	−0.25 *	NS	NS	NS	NS	NS	0.24 *	NS
INSL3/LH	75	NS	NS	NS	NS	NS	NS	NS	NS	NS	NS	NS	NS	NS

* *p* < 0.5, ** *p* < 0.01, *** *p* < 0.001; NS—not significant; N—number of cases; Abbreviations: A—after surgery; B—before surgery; Testicular parameters: TAI— testicular atrophy index; TAI-1—undescended testes in UCT and IAT group, bigger testes in BCT group compared to the healthy testes in UCT group; TAI-2—smaller testes in BCT group compared to the healthy testes in UCT group; TGP—testicular growth percentage; TGP-1—descended testes in UCT and IAT group, bigger testes in BCT group; TGP-2—undescended testes in UCT and IAT groups, smaller testes in BCT group; mean TGP—mean of both testes; TV—testicular volume; TV-1—descended testis in UCT and IAT groups, bigger testes in BCT group; TV-2—undescended testes in UCT and IAT groups, smaller testes in BCT group; mean TV—mean of both testes; Hormones: AMH—anti-Mȕllerian hormone, DHT—dihydrotestosterone, E2—estradiol, FSH—follicle-stimulating hormone, Inh B—inhibin B, INSL3—insulin like protein 3, LH—luteinizing hormone, T—testosterone.

**Table 4:** Spearman’s rank correlations (rs) between serum hormonal levels in boys from the control group and boys with undescended testes (UDT).

**Control Group**									
**Hormone**	**N**	**FSH**	**LH**	**T**	**E2**	**DHT**	**Inh B**	**AMH**	**INSL3**
FSH	51		NS	NS	NS	NS	NS	−0.30 *	NS
LH	51			0.71 ***	0.38 **	0.42 **	NS	NS	NS
T	51				0.28 *	0.53 ***	NS	NS	NS
E2	51					NS	NS	0.47 ***	NS
DHT	46						NS	NS	NS
Inh B	51							NS	0.41 **
AMH	51								NS
INSL 3	46								
**UDT group**									
**Hormone**	**N**	**FSH**	**LH**	**T**	**E2**	**DHT**	**Inh B**	**AMH**	**INSL3**
FSH	90		0.26 *	NS	NS	NS	NS	NS	NS
LH	90			NS	NS	NS	0.31 **	NS	0.32 **
T	90				NS	0.46 ***	NS	NS	NS
E2	90					NS	NS	0.40 ***	NS
DHT	86						NS	NS	NS
Inh B	83							0.22 *	0.41 ***
AMH	83								NS
INSL3	76								

* *p* < 0.5, ** *p* < 0.01, *** *p* < 0.001; NS—not significant; N—number of cases; Abbreviations: AMH—anti-Mȕllerian hormone, DHT—dihydrotestosterone, E2—estradiol, FSH—follicle-stimulating hormone, Inh B—inhibin B, INSL3—insulin like protein 3, LH—luteinizing hormone, and T—testosterone.

## Data Availability

The data presented in this study are available on reasonable request from the corresponding author.

## Supplementary Materials

The following supporting information can be downloaded at: https://www.mdpi.com/article/10.3390/jcm14010073/s1. Table S1a. Comparisons of hormone levels between UDT and control groups in the studied prepubertal boys below and above the 6th year of age. Table S1b. Spearman’s rank correlations (rs) between serum hormonal levels and age in boys from the control and UDT groups. Table S2a. Comparisons of testicular parameters in boys with UDT below and above the 6th year of age. Table S2b. Spearman’s rank correlations (rs) between testicular parameters and age in boys with UDT, and in different clinical types of undescended testes: unilateral canalicular (UCT), bilateral canalicular (BCT), and intra-abdominal (IAT). Table S3a. Spearman’s rank (rs) correlations between testicular parameters and serum hormonal levels in boys with UDT: unilateral canalicular (UCT), bilateral canalicular (BCT), and intra-abdominal (IAT). Table S3b. Spearman’s correlations (rs) between testicular parameters and serum hormonal levels in boys with UDT below and above the 6th year of age. Table S4a. Spearman’s rank correlations (rs) between serum hormonal levels in boys from the control group below and above the 6th year of age. Table S4b. Spearman’s rank correlations (rs) between serum hormonal levels in boys with UDT below and above the 6th year of age.
