# Supplementary material for: Hormonal Function of Undescended Testes Before Orchidopexy in Prepubertal Boys
Source: J Clin Med. 2024 Dec 27;14(1):73. doi: 10.3390/jcm14010073 (PMC11721048; doi:10.3390/jcm14010073)
Supplement: Supplementary file 1 [file jcm-14-00073-s001.zip › Table S1a.pdf]

Table S1a. Comparisons of hormones levels between UDT and Control groups in the studied prepubertal boys below and above the 6th year of age.

Hormones	<6. year		≥6. year	
	**Control<br>N=36**	**UDT<br>N=72**	**Control<br>N=21**	**UDT<br>N=18**
	**Mean±SD<br>Median<br>Min-Max**	**Mean±SD<br>Median<br>Min-Max**	**Mean±SD<br>Median<br>Min-Max**	**Mean±SD<br>Median<br>Min-Max**
FSH (U/l)	0.9±0.3<br>0.7<br>0.7-1.6	**1.3±0.8** [a]<br>**1.0**<br>**0.7-4.1**	**1.2±0.6**[c]<br>**1.1**<br>**0.7-2.7**	1.1 ±0.6<br>0.9<br>0.2-2.6
LH (U/l)	0.2±0.1<br>0.2<br>0.2-0.5	**0.4±0.4** [a]<br>**0.2**<br>**0.2-2.1**	**0.5±0.4** [c]<br>**0.3**<br>**0.2-1.7**	**0.2±0.1** [b, d]<br>**0.2**<br>**0.2-0.5**
T (nmol/l)	0.2±0.0<br>0.2<br>0.2-0.3	0.2±0.0<br>0.2<br>0.2-0.3	**0.3±0.2**[c]<br>**0.2**<br>**0.2-0.8**	**0.3±0.2** [d]<br>**0.2**<br>**0.2-0.7**
E2 (pmol/l)	40.2±12.2<br>37.9<br>23.3-67.0	45.9±19.4<br>41.1<br>23.0-105.2	41.4±20.1<br>35.1<br>23.3-99.6	39.1±11.0<br>42.9<br>23.3-55.1
DHT (pg/ml)	105.6±121.4<br>64.0<br>17.0-500.0	78.1.±84.0<br>55.0<br>10.0-469.0	**185.8±145.2**[c]<br>**172.0**<br>**9.0-464.0**	**177.3±130.7**[d]<br>**150.5**<br>**35.0-438.0**
Inh B (pg/ml)	133.8±84.0<br>137.9<br>3.1-344.4	122.3±72.5<br>103.8<br>10.1-322.9	115.3±66.8<br>101.0<br>24.8-228.8	**67.0±45.9** [b, d]<br>**61.0**<br>**6.7 -146.7**
AMH (ng/ml)	97.0±76.9<br>82.0<br>13.6-240.0	97.6±77.6<br>92.0<br>12.6-335.0	73.8±61.0<br>62.0<br>13.0-198.0	85.4±78.0<br>81.0<br>13.6 -287.0
INSL3 (pg/ml)	17.3±.13.8<br>13.9<br>5.6-75.0	19.1±7.6<br>20.6<br>5.8-32.1	15.2±.9.8<br>11.2<br>5.7-33.9	**12.9±.7.1**[d]<br>**10.5**<br>**5.8-25.1**
T/LH	0.8 ±0.1<br>0.8<br>0.4-0.8	**0.6±0.3**[a]<br>**0.8**<br>**0.1-1.5**	0.8±0.4<br>0.8<br>0.2-1.7	**1.2±0.6** [b, d]<br>**0.8**<br>**0.5-3.0**
Inh B/FSH	168.1±126.6<br>134.6<br>4.7-521.8	**120.2±102.4** [a]<br>**97.1**<br>**5.9-489.2**	109.4±72.9<br>67.8<br>34.9-251.2	95.5±136.1<br>59.2<br>4.1-594.9
AMH/FSH	129.7±110.9<br>97.7<br>10.5-363.6	91.8±89.4<br>76.5<br>6.1-384.4	73.8±71.7<br>60.4<br>5.2-300.0	94.8±86.1<br>74.8<br>8.9-337.5
Inh B/AMH	3.6±4.5<br>1.4<br>0.0-18.7	3.2±4.4<br>1.1<br>0.1-23.4	3.9±4.7<br>2.0<br>0.2-17.4	**1.4±1.6** [b]<br>**0.7**<br>**0.1-7.0**
INSL3/LH	75.7±62.9<br>53.5<br>21.9-347.2	61.1±36.2<br>50.9<br>4.9-148.1	**42.4±29.1**[c]<br>**33.1**<br>**4.9-108.2**	47.8±34.1<br>36.1<br>17.0-98.1

$p < 0.05$, a—Control (<6 y.) vs UDT (< 6 y.); b—Control (≥6 y.) vs UDT (≥6 y.); c—Control (<6 y.) vs Control (≥6 y.); d—UDT (<6 y.) vs UDT (≥6 y.); U Mann-Whitney test. Statistically significant data are in bold. Abbreviations: AMH—antimüllerian hormone, DHT—dihydrotestosterone, E2—estradiol, FSH—follicle stimulating hormone, Inh B—inhibin B, INSL 3—insulin like protein 3, LH—luteinizing hormone, N—number of cases, T—testosterone.
