# Supplementary material for: Hormonal Function of Undescended Testes Before Orchidopexy in Prepubertal Boys
Source: J Clin Med. 2024 Dec 27;14(1):73. doi: 10.3390/jcm14010073 (PMC11721048; doi:10.3390/jcm14010073)
Supplement: Supplementary file 1 [file jcm-14-00073-s001.zip › Table S1b.pdf]

Table S1b. Spearman's rank correlations (rs) between serum hormonal levels and age in boys from the Control and UDT groups

	**Control group**		**UDT group**	
**Parameter**	**N**	**Age**	**N**	**Age**
FSH	57	0.34**	90	NS
LH	57	0.35**	90	NS
T	57	0.59***	90	0.25*
E2	57	NS	90	NS
DHT	52	0.34*	85	0.38**
Inh B	51	NS	82	-0.49***
AMH	51	-0.29*	82	NS
INSL3	48	NS	75	-0.30**
T/LH	51	NS	90	NS
Inh B/FSH	51	-0.36**	83	-0.38***
AMH/FSH	51	-0.31*	83	NS
Inh B/AMH	51	NS	83	-0.25*
INSL3/LH	48	-0.40**	75	NS

*p <0.5, **p<0.01, ***p<0.001; Abbreviations: AMH—antimüllerian hormone (ng/ml), DHT—dihydrotestosterone (pg/ml), E2—estradiol (pmol/l), FSH—follicle stimulating hormone (U/l), Inh B—inhibin B (pg/ml), INSL3—insulin like protein 3 (pg/ml), LH—luteinizing hormone (U/l), N—number of cases, NS—not significant, T—testosterone (nmol/l), UDT—group of boys with undescended testes.
