# Supplementary material for: Hormonal Function of Undescended Testes Before Orchidopexy in Prepubertal Boys
Source: J Clin Med. 2024 Dec 27;14(1):73. doi: 10.3390/jcm14010073 (PMC11721048; doi:10.3390/jcm14010073)
Supplement: Supplementary file 1 [file jcm-14-00073-s001.zip › Table S2a.pdf]

Table S2a. Comparisons of testicular parameters in boys with UDT below and above the 6th year of age.

Testicular parameters	<6 year	≥6 year
	UDT<br>N=72	UDT<br>N=18
	Mean±SD<br>Median<br>Min-Max	Mean±SD<br>Median<br>Min-Max
Unilateral n (%)<br>Bilateral n (%)	63 (88%)<br>9 (12%)	14 (78%)<br>4 (22%)
TV-1 (B)	0.7±0.3<br>0.7<br>0.3-1.2	**1.4±0.3*****<br>**1.4**<br>**0.8-2.2**
TV-2 (B)	0.5±0.2<br>0.5<br>0.5-1.0	**1.1±0.4*****<br>**1.3**<br>**0.5-1.7**
Mean TV (B)	0.6±0.2<br>0.7<br>0.2-1.2	**1.2±0.3*****<br>**1.3**<br>**0.8 -2.0**
TAI-1 (B)	25.0±15.8<br>22.2<br>0-75.0	**18.1±17.2 ***<br>**12.9**<br>**0-68.7**
TAI-2 (B)	36.8±22.6<br>25.6<br>16.3-74.4	25.1±18.1<br>17.4<br>13.3-52.0
TV-1 (A)	1.0±0.3<br>1.0<br>0.5-2.0	**1.7±0.6*****<br>**1.8**<br>**0.8-3.5**
TV-2 (A)	0.8±0.3<br>0.8<br>0.1-1.7	**1.5±0.6*****<br>**1.7**<br>**0.5-2.9**
Mean TV (A)	0.9±0.2<br>0.9.<br>0.5-1.9	**1.6±0.6*****<br>**1.7**<br>**0.7-3.2.**
TAI-1 (A)	17.6±11.4<br>16.7<br>0.0-60.0	15.6±16.4<br>11.8<br>-5.3-63.2
TAI-2 (A)	37.0±31.8<br>29.0<br>-8.0-88.3	19.6±25.6<br>25.6<br>-7.4-51.0
TGP-1	56.0±49.6<br>25.0.<br>0.0-200.0	**24.2±19.4****<br>**20.5**<br>**0.0-78.6**
TGP-2	75.4±68.5<br>60.0<br>-54.8-300.0	**32.7±23.9***<br>**34.5**<br>**-28.6-70.6**
Mean TGP	62.8±51.6<br>50.0<br>-5.7-200.0	**27.4±20.2***<br>**23.3**<br>**-13.3-74.1**

*p <0.05; **p<0.01; ***p<0.001; U Mann-Whitney test. Statistically significant data are in bold. Abbreviations: A—after surgery, B—before surgery, N—number of cases; TAI —testicular atrophy index (%); TAI-1—undescended testis in UCT and IAT group, bigger testis in BCT group compared to the healthy testis in UCT group; TAI-2—smaller testis in BCT group compared to the healthy testis in UCT group; TGP—testicular growth percentage (%); TGP-1—descended testis in UCT and IAT group, bigger testis in BCT group; TGP-2—undescended testis in UCT and IAT group, smaller testis in BCT group; Mean TGP – mean of both testes; TV—testicular volume (cm^3); TV-1—descended testis in UCT and IAT group, bigger testis in BCT group; TV-2—undescended testis in UCT and IAT group, smaller testis in BCT group; Mean TV—mean of both testes.
