# Supplementary material for: Hormonal Function of Undescended Testes Before Orchidopexy in Prepubertal Boys
Source: J Clin Med. 2024 Dec 27;14(1):73. doi: 10.3390/jcm14010073 (PMC11721048; doi:10.3390/jcm14010073)
Supplement: Supplementary file 1 [file jcm-14-00073-s001.zip › Table S2b.pdf]

Table S2b. Spearman's rank correlations (rs) between testicular parameters and age in boys with UDT, and in different clinical types of undescended testes: unilateral canalicular (UCT), bilateral canalicular (BCT) and intra-abdominal (IAT)

Testicular parameters	UDT N=90	UCT N=65	BCT N=13	IAT N=12
TV-1 (B)	0.84***	0.82***	0.74**	NS
TV-2 (B)	0.78***	0.73***	0.66*	NS
Mean TV (B)	0.83***	0.81***	0.71**	0.62*
TAI-1 (B)	-0.29**	NS	NS	NS
TAI-2 (B)	NS	-	NS	-
TV-1 (A)	0.74***	0.67***	0.91***	0.77**
TV-2 (A)	0.62***	0.59***	NS	NS
Mean TV (A)	0.70***	0.67***	0.64*	0.60**
TAI-1 (A)	NS	NS	NS	NS
TAI-2 (A)	NS	-	NS	-
TGP-1	-0.55***	-0.49***	NS	NS
TGP-2	-0.58***	-0.44***	NS	NS
Mean TGP	-0.56***	-0.47***	NS	NS

*p <0.05; **p<0.01; ***p<0.001; Abbreviations: A—after surgery; B—before surgery; N—number of cases; NS- not significant; TAI—testicular atrophy index (%); TAI-1—undescended testis in UCT and IAT group, bigger testis in BCT group compared to the healthy testis in UCT group; TAI-2—smaller testis in BCT group compared to the healthy testis in UCT group; TGP—testicular growth percentage (%); TGP-1—descended testis in UCT and IAT group, bigger testis in BCT group; TGP-2—undescended testis in UCT and IAT group, smaller testis in BCT group; Mean TGP—mean of both testes; TV—testicular volume (cm^3); TV-1—descended testis in UCT and IAT group, bigger testis in BCT group; TV-2—undescended testis in UCT and IAT group, smaller testis in BCT group; Mean TV—mean of both testes.
