# Supplementary material for: Hormonal Function of Undescended Testes Before Orchidopexy in Prepubertal Boys
Source: J Clin Med. 2024 Dec 27;14(1):73. doi: 10.3390/jcm14010073 (PMC11721048; doi:10.3390/jcm14010073)
Supplement: Supplementary file 1 [file jcm-14-00073-s001.zip › Table S3a.pdf]

Table S3a. Spearman's rank (rs) correlations between testicular parameters and serum hormonal levels in boys with UDT: unilateral canalicular (UCT), bilateral canalicular (BCT) and intra-abdominal (IAT).

Parameter	N	TV-1 (B)	TV-2 (B)	Mean TV (B)	TAI-1 (B)	TAI-2 (B)	TV-1 (A)	TV-2 (A)	Mean TV (A)	TAI-1 (A)	TAI-2 (A)	TGP-1	TGP-2	Mean TGP
**UCT**														
FSH	65	NS	NS	NS	NS	-	NS	NS	NS	NS	-	NS	NS	NS
LH	65	-0.25*	NS	NS	NS	-	NS	NS	NS	NS	-	NS	NS	NS
T	65	0.39**	NS	0.34**		-	0.41***	0.30*	0.38**	NS	-	NS	NS	NS
E2	65	NS	NS	NS	NS	-	NS	NS	NS	NS	-	NS	NS	NS
DHT	62	0.36**	0.36**	0.37**	NS	-	NS	NS	NS	NS	-	-0.28*	-0.30*	-0.27*
Inh B	60	-0.53***	-0.42***	-0.52***	NS	-	-0.44***	-0.36**	-0.44***	NS	-	0.38**	0.29*	0.34**
AMH	69	NS	NS	NS	NS	-	NS	NS	NS	NS	-	NS	NS	NS
INSL 3	57	-0.35**	NS	-0.31*	NS	-	-0.29*	NS	-0.26*	NS	-	NS	NS	NS
T/LH	65	0.42***	0.31*	0.39**	NS	-	0.40***	0.32**	0.39**	NS	-	NS	NS	NS
Inh B/FSH	60	-0.42***	-0.36**	-0.43***	NS	-	-0.38**	-0.31*	-0.37**	NS	-	NS	NS	NS
AMH/FSH	60	NS	NS	NS	NS	-	NS	NS	NS	NS	-	NS	NS	NS
Inh B/AMH	60	-0.40***	-0.37***	- 0.42***	NS	-	- 0.28*	NS	- 0.26*	NS	-	NS	0.31*	0.28*
INSL3/LH	57	NS	NS	NS	NS	-	NS	NS	NS	NS	-	NS	NS	NS
**BCT**														
FSH	13	NS	NS	NS	NS	NS	NS	NS	NS	NS	NS	NS	NS	NS
LH	13	NS	NS	NS	NS	NS	NS	NS	NS	0.62*	NS	NS	NS	NS
T	13	NS	NS	NS	NS	NS	NS	NS	NS	NS	NS	NS	NS	NS
E2	12	NS	NS	NS	NS	NS	NS	NS	NS	NS	NS	NS	NS	NS
DHT	12	NS	NS	NS	NS	NS	NS	NS	NS	NS	NS	NS	NS	NS

Inh B	13	NS	NS	NS	NS	NS	NS	NS	NS	0.70**	NS	-0.58*	NS	NS
AMH	13	NS	NS	NS	NS	NS	NS	NS	NS	NS	NS	NS	NS	NS
INSL 3	11	NS	NS	NS	NS	NS	-0.81**	NS	NS	NS	NS	NS	NS	NS
T/LH	13	NS	NS	NS	NS	NS	NS	NS	NS	-0.67*	NS	NS	NS	NS
Inh B/FSH	13	NS	NS	NS	NS	NS	NS	NS	NS	NS	NS	NS	NS	NS
AMH/FSH	13	NS	NS	NS	NS	NS	NS	NS	NS	NS	NS	NS	NS	NS
Inh B/AMH	13	NS	NS	NS	NS	NS	NS	NS	NS	NS	NS	NS	NS	NS
INSL3/LH	11	NS	NS	NS	NS	NS	NS	-0.62**	NS	NS	NS	NS	NS	NS
**IAT**														
FSH	12	NS	NS	NS	NS	-	NS	NS	NS	NS	-	NS	NS	NS
LH	12	NS	NS	NS	NS	-	NS	NS	NS	NS	-	NS	NS	NS
T	12	NS	NS	NS	NS	-	NS	NS	NS	NS	-	NS	NS	NS
E2	12	NS	NS	NS	NS	-	NS	NS	NS	NS	-	NS	NS	NS
DHT	11	NS	NS	NS	NS	-	NS	NS	NS	NS	-	NS	NS	NS
Inh B	9	NS	NS	NS	NS	-	NS	0.74*	0.73*	NS	-	NS	NS	NS
AMH	9	NS	0.73*	NS	NS	-	NS	0.76*	0.81**	NS	-	NS	NS	NS
INSL 3	7	NS	NS	NS	NS	-	NS	NS	NS	NS	-	-0.79*	NS	-0.77*
T/LH	12	NS	NS	NS	NS	-	NS	NS	NS	NS	-	NS	NS	NS
Inh B/FSH	9	NS	NS	NS	NS	-	NS	NS	NS	NS	-	NS	NS	NS
AMH/FSH	9	NS	NS	NS	NS	-	NS	NS	NS	NS	-	NS	NS	NS
Inh B/AMH	9	NS	NS	NS	NS	-	NS	NS	NS	NS	-	NS	NS	NS
INSL3/LH	**7**	NS	NS	NS	NS	-	NS	NS	NS	NS	-	-0.79*	NS	-0.77*

*p <0.05; **p<0.01; ***p<0.001. Abbreviations: A—after surgery; B—before surgery; N- number of cases; NS—not significant; Testicular parameters: TAI—testicular atrophy index (%); TAI-1—undescended testis in UCT and IAT group, bigger testis in BCT group compared to the healthy testis in UCT group; TAI-2—smaller testis in BCT group compared to the healthy testis in UCT group; TGP – testicular growth

percentage (%); TGP-1—descended testis in UCT and IAT group, bigger testis in BCT group; TGP-2—undescended testis in UCT and IAT group, smaller testis in BCT group; Mean TGP – mean of both testes; TV—testicular volume (cm3); TV-1—descended testis in UCT and IAT groups, bigger testis in BCT group; TV-2—undescended testis in UCT and IAT groups, smaller testis in BCT group; Mean TV—mean of both testes; Hormones: AMH—antimùllerian hormone, DHT—dihydrotestosterone, E2—estradiol, FSH—follicle stimulating hormone, Inh B—inhibin B, INSL3 – insulin like protein 3, LH—luteinizing hormone, T—testosterone.
