# Supplementary material for: Hormonal Function of Undescended Testes Before Orchidopexy in Prepubertal Boys
Source: J Clin Med. 2024 Dec 27;14(1):73. doi: 10.3390/jcm14010073 (PMC11721048; doi:10.3390/jcm14010073)
Supplement: Supplementary file 1 [file jcm-14-00073-s001.zip › Table S3b.pdf]

Table S3b. Spearman's rank correlations (rs) between testicular parameters and serum hormonal levels in boys with UDT below and above the 6th year of age.

**<6 years**														
**Parameter**	**N**	**TV-1 (B)**	**TV-2 (B)**	**Mean TV (B)**	**TAI-1 (B)**	**TAI-2 (B)**	**TV-1 (A)**	**TV-2 (A)**	**Mean TV (A)**	**TAI-1 (A)**	**TAI-2 (A)**	**TGP-1**	**TGP-2**	**Mean TGP**
FSH	72	NS	NS	NS	NS	NS	NS	NS	NS	NS	NS	NS	NS	NS
LH	72	NS	NS	NS	NS	NS	NS	NS	NS	NS	NS	NS	NS	NS
T	72	NS	NS	NS	NS	NS	NS	NS	NS	NS	NS	NS	NS	NS
E2	72	NS	NS	NS	NS	NS	NS	NS	NS	NS	NS	NS	NS	NS
DHT	69	NS	NS	NS	NS	NS	NS	NS	NS	NS	NS	NS	NS	NS
Inh B	64	-0.34**	NS	-0.32*	NS	NS	-0.31*	NS	NS	NS	NS	NS	NS	NS
AMH	64	NS	NS	NS	NS	NS	NS	NS	NS	NS	NS	NS	NS	NS
INSL3	62	NS	NS	NS	NS	NS	NS	NS	NS	NS	NS	NS	NS	NS
T/LH	72	NS	NS	NS	NS	NS	NS	NS	NS	NS	NS	NS	NS	0.24*
Inh B/FSH	64	-0.29*	NS	-0.27*	NS	NS	-0.28*	NS	NS	NS	NS	NS	NS	NS
AMH/FSH	64	NS	NS	NS	NS	NS	NS	NS	NS	NS	NS	NS	NS	NS
Inh B/AMH	64	NS	NS	NS	NS	NS	NS	NS	NS	NS	NS	NS	0.25*	NS
INSL3/LH	62	NS	NS	NS	NS	NS	NS	NS	NS	NS	NS	NS	NS	NS
**≥6 years**														
FSH	18	NS	NS	NS	NS	NS	NS	NS	NS	NS	NS	NS	NS	NS
LH	18	NS	NS	NS	NS	NS	NS	NS	NS	NS	NS	NS	NS	NS
T	18	0.55*	NS	NS	NS	NS	0.54*	NS	NS	NS	NS	NS	NS	NS
E2	17	NS	NS	NS	NS	NS	NS	NS	NS	NS	NS	NS	NS	NS
DHT	16	NS	NS	NS	NS	NS	NS	NS	NS	NS	NS	NS	NS	NS
Inh B	18	NS	NS	NS	NS	NS	NS	NS	NS	NS	NS	NS	NS	NS
AMH	18	NS	0.51*	0.48*	NS	NS	NS	0.51*	0.49*	NS	NS	NS	NS	NS

INSL 3	13	NS	NS	NS	NS	NS	NS	NS	NS	NS	NS	NS	NS	NS
T/LH	18	NS	NS	NS	NS	NS	0.48*	NS	NS	NS	NS	NS	NS	NS
Inh B/FSH	18	NS	NS	NS	NS	NS	NS	NS	NS	NS	NS	NS	NS	NS
AMH/FSH	18	NS	NS	NS	NS	NS	NS	NS	NS	NS	NS	NS	NS	NS
Inh B/AMH	18	NS	NS	NS	NS	NS	NS	NS	NS	NS	NS	NS	NS	NS
INSL3/LH	13	NS	NS	NS	NS	NS	NS	NS	NS	NS	NS	NS	NS	NS

*p <0.5, **p<0.01, ***p<0.001; Abbreviations: A—after surgery; B—before surgery; N—number of cases; NS—not significant; Testicular parameters: TAI—testicular atrophy index (%); TAI-1—undescended testis in UCT and IAT group, bigger testis in BCT group compared to the healthy testis in UCT group; TAI-2—smaller testis in BCT group compared to the healthy testis in UCT group; TGP—testicular growth percentage (%); TGP-1—descended testis in UCT and IAT group, bigger testis in BCT group; TGP-2—undescended testis in UCT and IAT group, smaller testis in BCT group; Mean TGP—mean of both testes; TV—testicular volume; TV-1—descended testis in UCT and IAT groups, bigger testis in BCT group; TV-2—undescended testis in UCT and IAT groups, smaller testis in BCT group; Mean TV—mean of both testes; Hormones: AMH—antimüllerian hormone, DHT—dihydrotestosterone, E2—estradiol, FSH—follicle stimulating hormone, Inh B—inhibin B, INSL3—insulin like protein 3, LH—luteinizing hormone; T—testosterone.
