# Supplementary material for: Hormonal Function of Undescended Testes Before Orchidopexy in Prepubertal Boys
Source: J Clin Med. 2024 Dec 27;14(1):73. doi: 10.3390/jcm14010073 (PMC11721048; doi:10.3390/jcm14010073)
Supplement: Supplementary file 1 [file jcm-14-00073-s001.zip › Table S4a.pdf]

Table S4a. Spearman's rank correlations (rs) between serum hormonal levels in boys from Control group below and above 6th year of age.

< 6 years									
**Hormone**	**N**	**FSH**	**LH**	**T**	**E2**	**DHT**	**Inh B**	**AMH**	**INSL3**
FSH	36		NS	NS	NS	NS	NS	-0.45*	NS
LH	36			0.45**	0.41**	NS	NS	NS	NS
T	36				NS	NS	NS	NS	NS
E2	36					NS	NS	0.45**	NS
DHT	33						NS	NS	NS
Inh B	30							NS	0.45**
AMH	30								NS
INSL 3	30								
**≥ 6 years**									
**Hormone**	**N**	**FSH**	**LH**	**T**	**E2**	**DHT**	**Inh B**	**AMH**	**INSL3**
FSH	21		NS	NS	NS	NS	NS	NS	NS
LH	21			0.68**	0.52**	0.47**	NS	NS	NS
T	21				0.63***	0.85**	NS	NS	NS
E2	21					0.63**	NS	0.50***	NS
DHT	19						NS	NS	NS
Inh B	21							NS	NS
AMH	21								NS
INSL 3	18								

*p <0.5, **p<0.01, ***p<0.001; Abbreviations: AMH—antimüllerian hormone, DHT—dihydrotestosterone, E2—estradiol, FSH—follicle stimulating hormone, Inh B—inhibin B, INSL3—insulin like protein 3, LH—luteinizing hormone, N—number of cases, NS—not significant, T—testosterone.
