# Supplementary material for: Hormonal Function of Undescended Testes Before Orchidopexy in Prepubertal Boys
Source: J Clin Med. 2024 Dec 27;14(1):73. doi: 10.3390/jcm14010073 (PMC11721048; doi:10.3390/jcm14010073)
Supplement: Supplementary file 1 [file jcm-14-00073-s001.zip › Table S4b.pdf]

Table S4b. Spearman's rank correlations (rs) between serum hormonal levels in boys with undescended testes (UDT) below and above the 6th year of age.

**<6 years**									
**Hormone**	**N**	**FSH**	**LH**	**T**	**E2**	**DHT**	**Inh B**	**AMH**	**INSL3**
FSH	72		0.25*	NS	NS	NS	NS	NS	NS
LH	72			NS	NS	NS	NS	NS	0.25*
T	72				NS	0.25*	NS	NS	NS
E2	72					NS	NS	0.40**	NS
DHT	69						NS	NS	NS
Inh B	64							NS	0.38**
AMH	64								NS
INSL 3	62								
**≥ 6 years**									
**Hormone**	**N**	**FSH**	**LH**	**T**	**E2**	**DHT**	**Inh B**	**AMH**	**INSL3**
FSH	18		NS	NS	NS	NS	NS	NS	NS
LH	18			NS	NS	NS	0.47**	0.51**	NS
T	18				NS	0.89***	NS	NS	NS
E2	17					NS	NS	NS	NS
DHT	16						NS	NS	NS
Inh B	18							0.50**	NS
AMH	18								NS
INSL 3	13								

*p <0.5, **p<0.01, ***p<0.001; *p <0.5, **p<0.01, ***p<0.001; Abbreviations: AMH—antimüllerian hormone, DHT—dihydrotestosterone, E2—estradiol, FSH—follicle stimulating hormone, Inh B—inhibin B, INSL3—insulin like protein 3, LH—luteinizing hormone, N—number of c ases, NS—not significant, T—testosterone.
